# The Polish version of the Düsseldorf Orthorexia Scale (PL-DOS) and its comparison with the English version of the DOS (E-DOS)

**DOI:** 10.1007/s40519-020-01025-z

**Published:** 2020-10-06

**Authors:** Anna Brytek-Matera

**Affiliations:** Institute of Psychology, University of Wroclaw, Dawida 1, 50-527 Wrocław, Poland

**Keywords:** Orthorexia nervosa, Eating behaviours, Prevalence, DOS

## Abstract

**Purpose:**

Although orthorexia nervosa, the fixation on health-conscious eating behaviour, was first described in the 90 s, there is no clear understanding whether existing ON measures are appropriate for its assessment. The objectives of the present study were to: (1) examine the psychometric properties of the Polish version of the DOS (PL-DOS) and to compare the PL-DOS with the English version of the DOS (E-DOS) as well as (2) evaluate the prevalence of ON among Polish university students and compare the prevalence rates of ON among Polish and U.S. students.

**Methods:**

Four-hundred and twelve students (77.2% female) with a mean age of 24.62 years (SD = 6.86) participated in the present study. All participants completed the Polish version of the Düsseldorf Orthorexia Scale (PL-DOS), the Eating Habits Questionnaire (EHQ) and the Eating Disorder Inventory (EDI).

**Results:**

Reliability analysis for the PL-DOS showed strong internal consistency with a Cronbach’s alpha coefficient of 0.840 and a coefficient omega of 0.840, 95% CI [0.808, 0.866]. Significant correlation coefficients were found between the PL-DOS and all subscales of the EHQ. Confirmatory factor analysis showed that the one-factor model had poor fit. Polish students had an ON prevalence rate of 6.6%, lower than that of U.S. students (8%).

**Conclusions:**

Our findings validate the use of the PL-DOS as an appropriate ON measure for a Polish population.

**Level of evidence:**

Level V, cross-sectional descriptive study.

## Introduction

In recent years, the number of different types of dietary styles has been increasing. The need to eat “clean” and unprocessed foods is becoming more popular among many people and young people in particular [1]. In developed Western societies, great attention is being paid to the individual responsibility for one’s health (“new health consciousness”), called “healthism” [2, 3]. Individuals try to reach “perfect health” through individual discipline and moral conduct [4]. Regular exercise and healthy eating habits are the pillars of this concept which has become part of today’s consumer culture. However, even self-destructive habits may be adopted to conform with what people consider “perfect health”. There is a thin line between following a certain diet or exercise program and adhering to a dietary and exercise regimen that causes great discomfort when it cannot be followed. Therefore, the problem of eating-related disorders is growing more severe in contemporary society. In some individuals, interest in healthy attitudes and behaviours towards food may show signs of obsession [5]. Preoccupation with “healthful” eating may contribute to orthorexia nervosa (ON): “a fixation on eating healthy food” [6; p. 9] according to the individual’s own criteria (including but not limited to, fixation on organic, and biologically clean products, dietary supplements, raw versus cooked foods, and high or low carbohydrate diets). Features include overly caring for one’s health, obtrusive thoughts on being healthy, lack of flexibility in diet, long-term diet planning and negative impact of food choices on quality of life.

According to the proposed diagnostic criteria by Dunn and Bratman [7], individuals suffering from ON are preoccupied with either affirmative or restrictive dietary practices focusing on concerns regarding the quality and composition of meals (“healthy foods”) with strict avoidance of foods believed to be unhealthy. Dietary restrictions escalate over time and may cause the exclusion of entire food groups (e.g., foods containing any fat, preservatives, food-additives, animal products, or other ingredients considered by the subject to be unhealthy) and excessive amounts of time spent on reading about, acquiring and/or preparing specific types of foods based on their perceived quality and composition. Individuals suffering from ON pursue their healthy eating preoccupation by following a strict diet based on food quality (and not quantity) and composition. Violations of the restrictive dietary rules may result in extreme emotional distress with feelings of guilt, shame, worries and/or anxiety. Nutritional deficiencies may lead to significant weight loss, malnutrition, and/or physical health complications and psychosocial impairments in social, vocational, and/or academic functioning that may result from the other-diagnostic criteria [7].

ON has been considered as both an obsessive–compulsive spectrum disorder and an eating disorder such as anorexia nervosa (AN) [8]. ON could be treated as an eating disorder, because it is closely associated with the excessive immersion in different aspects of eating behaviour [9] due to a restrictive diet, perfectionistic tendencies [10], co-occurrence of anxiety and a need for control [11] and the presence of ritual food preparation [12]. Nevertheless, AN patients seem more obsessed about quantity of food intake, body image, and physical appearance [13], while ON patients seem to be more preoccupied with the quality of food [14]. In addition, individuals with ON tend to concentrate on providing maximally healthy and “clean” foods instead of controlling body weight and being dissatisfied with their body image [10]. These results provide evidence that ON is not associated with dysfunctional attitudes towards the body, which suggests that ON should not be treated as a classical eating disorder. Moreover, in the clinical expression of ON, there is a lack of the most characteristic traits of an eating disorder: an excessive preoccupation with losing body weight, strong anxiety about gaining weight, and inaccurate assessment of body size [15].

Although the amount of ON measures has increased in the last several years, to date, it remains an open question whether existing ON measures are appropriate for the assessment of ON. To date, the ORTO-15 is probably the most commonly used self-report measure of ON [16]. The ORTO-15 has good predictive validity [17], nevertheless the lack of basic psychometric properties and the internal consistency has been criticized [18]. Psychometric flaws of the ORTO-15 indicate that this method should be replaced by another measurement. The Eating Habits Questionnaire (EHQ) [13], evaluates cognitions (knowledge of healthy eating), behaviours (problems associated with healthy eating) and feelings (feeling positively about healthy eating) regarding an extreme focus on healthy eating. The EHQ shows satisfactory internal consistency estimates and test–retest reliability [13]. The Düsseldorf Orthorexia Scale (DOS) [19], a screening instrument, assesses orthorexic eating behaviour. The DOS reports good internal consistency, good construct validity as well as good test–retest reliability. Recent research [18] has shown that both the EHQ and DOS are internally reliable self-report instruments. They have a unidimensional structure (higher order for the EHQ), good internal reliability and sum scores are highly correlated with each other, pointing out that they measure the same construct [20]. In contrast, the ORTO-15 is internally unreliable, its factor structure has an unacceptable model fit and the scale has low internal reliability and shows medium-sized correlations with the EHQ and the DOS, implying that it measures a different construct or captures ON less precisely. Thus, researchers studying ON should consider using the EHQ or the DOS [18, 21].

The Düsseldorf Orthorexia Scale has been translated into English [22], Spanish [23], and Chinese [24] so far. Due to the lack of a well-defined criterion for determining an optimal cut-off value, estimates of ON prevalence (using the ORTO-15) vary considerably. Taking into consideration only the DOS with its proposed cutoff (score ≥ 30) the prevalence of ON in a representative German sample was estimated to be 6.9% [25]. Additional findings have revealed that the prevalence rate of ON among university students was 3.3% in a German sample [26], 7.8% in a Chinese sample [24], 8% in a U.S. sample [22] and 10.5% in a Spanish sample [23].

Given the lack of a Polish version of the DOS and the inconsistency within pooled prevalence estimates of ON based on the DOS, the aims of the present study were to: (1) examine the psychometric properties of the Polish version of the DOS (PL-DOS) and to compare PL-DOS English version of the DOS (E-DOS) as well as (2) evaluate the prevalence of ON according to the DOS among Polish university students and compare prevalence rates of ON among Polish and U.S. students.

The following hypotheses were put forward:

H1. Individual items of the PL-DOS will show positive correlations with knowledge of healthy eating, problems associated with healthy eating and feeling positive about healthy eating measured by the EHQ.

H2. Individual items of the PL-DOS will show low correlations with eating disorder psychopathology and related features measured by the EDI.

H3. The PL-DOS will demonstrate good internal consistency.

## Materials and methods

### Participants and procedure

A sample of 318 female (77.2%) and 94 male (22.8%) (*N*_total_ = 412) university students participated in the present study. The mean age of the sample was 24.62 (SD = 6.86, range 18–64) years. The average Body Mass Index (BMI; derived from self-reported height and weight) was 23.12 kg/m2 (SD = 4.65, range 16.20–57.60). Among all participants, 6.9% were underweight (below 18.5 kg/m^2^), 68.9% had a normal weight (from 18.5 to 24.99 kg/m^2^), 16.7% were overweight (from 25.0 to 29.99 kg/m^2^) and 7.2% were obese (≥ 30.0 kg/m^2^). 60% of the participants declared to follow an omnivorous diet, 10.4% declared to follow a vegetarian diet (2.9% = fruitarian diet, 1.7% = lacto-ovo-vegetarian diet, 1.2% = lacto-vegetarian diet, 1.9% = ovovegetarian diet, and 2.7% = vegan diet) and 9.7% of the participants declared to follow other diets (e.g., 1.7% = pesco-vegetarian diet).

A convenience sample of university students was recruited to take part in an online survey. Participants received notice about the research with the announcement including the online link to the study. Interested university students were invited to visit a website that directed them to the consent form, information form (purpose of the current study, anonymity, voluntariness of consent to research), and questionnaires. There was no financial compensation for participating in the study. The study was approved by the SWPS University of Social Sciences and Humanities Human Research Ethics Committee (no. WKEB59/05/2019).

### Measures

The Düsseldorf Orthorexia Scale [19] is a unidimensional measure for assessing and screening ON. It is composed of 10 items. The maximum score is 40. A score of 30 or higher indicates the presence of ON. A score between 25 and 29 identifies a risk of developing ON, while a total score of less than 25 demonstrates the absence of ON [26, 27]. The DOS shows good internal consistency (Cronbach’s alpha = 0.84) and good test–retest reliability (*r* = 0.67–0.79, *p* = 0.001) [19]. Taking into consideration the first aim of our study, we decided to use the same version of the DOS (English version of the DOS) that was used by Chard et al. [21] among a U.S. student sample. In the current study, the Polish version of the ten-item DOS (PL-DOS) was used after following standard translation procedures [28]. The DOS was translated from English to Polish using a standard forward–backward translation procedure. The English version of the DOS test was first translated into Polish (by two translators who independently translated the same questionnaire) and then back-translated into English (by a native English speaker without reference to the English original). In our translation procedure, we used the same standard as He et al.[24]: the back-translated version and the original English version were sent to two psychologists (experts in eating behaviour) for review. Based on their comments, we did minor modifications in the wording of the translation. The final version of the Polish translation was used in subsequent validation procedures. The Polish version of the DOS is presented in Appendix 1.

The Eating Habits Questionnaire [13] is a multidimensional measure for evaluating knowledge of healthy eating, problems associated with healthy eating and feeling positively about healthy eating. This 21-item self-report inventory, designed to measure a pathological fixation on healthy eating, displays good internal consistency (*α* = 0.82–0.90) and test–retest reliability (*r* = 0.72–0.81) [13]. In the current sample, Cronbach’s alpha coefficients of the EHQ subscales ranged from *α* = 0.77–0.86. In the present study, we used the Polish version of the EHQ [16].

The Eating Disorder Inventory [29] is a widely used self-report questionnaire that assesses the presence of eating disorder psychopathology and related features (drive for thinness, bulimia, body satisfaction, ineffectiveness, perfectionism, interpersonal distrust, interoceptive awareness and maturity fears). The Polish version of the EDI shows a satisfactory internal consistency (*α* = 0.65–0.92) [30]. In the current sample, Cronbach’s alpha coefficients of the EDI subscales ranged from *α* = 0.73–0.88.

### Statistical analyses

The statistical analyses were performed with IBM SPSS software version 26/R and IBM SPSS Amos version 3.6.2. The two-tailed level of significance was set at 0.05.

Cronbach’s alpha and the coefficient omega were used to assess the internal consistency of the PL-DOS. Item selectivity of the different PL-DOS items was assessed by calculating the corrected item-total correlation associations. In addition, to test concurrent validity, Pearson’s correlations of the DOS with the subscales of the EHQ and the subscales of the EDI were examined.

To confirm the latent structure of the PL-DOS, a first order confirmatory factor analysis (CFA) with asymptotically distribution-free estimates was used to assess the one-factor solution [19, 23] in the first place, and the second-order models with three [24] and five [22] first order factors in the second place. For evaluating the model fit, the following fit indicators with respective recommended cutoff values [31] were reported: the root mean square error of approximation (RMSEA; less than 0.06 indicating good fit) with its 90% confidence interval, comparative fit index (CFI; greater than 0.90 a reasonable fit), Tucker-Lewis Index (TLI; greater than 0.95 indicating good fit), and the overall model Chi square (*χ*^2^).

In the present study we tried to maximize data collection. Our solution of handling missing data in analysis were omitting variables which have many missing values and omitting participants who do not have complete data.

## Results

### Reliability and validity

Reliability analysis for the PL-DOS showed strong internal consistency with Cronbach’s alpha coefficient of 0.840 and coefficient omega of 0.840, 95% CI [0.808, 0.866].

Scale intercorrelations yielded an item to total score ranging from 0.40 to 0.63 for PL-DOS, indicating good item selectivity. Mean and standard deviation for each item of the PL-DOS including item selectivity (*r*) are presented in Table 1.

**Table 1:** Mean ± standard deviation of each item and item selectivity in the Polish 10-item DOS compared to E-DOS*

Item	Mean	SD	Item selectivity	Item selectivity from E-DOS [22]
1. Eating healthy food is more important to me than indulgence/enjoying the food	2.35	0.85	0.51	0.56
2. I have certain nutrition rules that I adhere to	2.72	0.96	0.40	0.60
3. I can only enjoy eating foods considered healthy	2.05	0.82	0.51	0.59
4. I try to avoid getting invited over to friends for dinner if I know that they do not pay attention to healthy nutrition	1.57	0.78	0.52	0.58
5. I like that I pay more attention to healthy nutrition than other people	2.09	0.93	0.57	0.59
6. If I eat something I consider unhealthy, I feel really bad	2.26	1.00	0.56	0.58
7. I have the feeling of being excluded by my friends and colleagues due to my strict nutrition rules	1.48	0.76	0.47	0.58
8. My thoughts constantly revolve around healthy nutrition and I organize my day around it	1.74	0.88	0.63	0.71
9. I find it difficult to go against my personal dietary rules	1.99	0.92	0.55	0.73
10. I feel upset after eating unhealthy foods	1.95	0.92	0.62	0.59

*The data from the original publication of the E-DOS [22]

### Construct validity

Significant correlation coefficients were found between the PL-DOS and the three subscales of the EHQ (Table 2). Correlation between the PL-DOS and the EDI showed low correlation coefficients ranging from 0.132 (perfectionism) to 0.311 (drive for thinness).

**Table 2:** Pearson correlations between the PL-DOS total score and EHQ and the EDI subscales in Polish sample compared to E-DOS*

	*PL-DOS* *r*	*PL-DOS* *p*	*E-DOS* [22] *r*	*E-DOS* [22] *p*
EHQ_Total score	0.506	** < 0.001**	0.762	< 0.001
EHQ_Knowledge of healthy eating	0.428	** < 0.001**	0.638	< 0.001
EHQ_Problems associated with healthy eating	0.473	** < 0.001**	0.729	< 0.001
EHQ_Feeling positively about healthy eating	0.381	** < 0.001**	0.478	< 0.001
EDI_Drive for thinness	0.311	** < 0.001**	0.483	< 0.001
EDI_Bulimia	0.196	** < 0.001**	0.245	< 0.001
EDI_Body dissatisfaction	0.096	0.053	0.190	< 0.001
EDI_Ineffectiveness	0.056	0.253	0.138	< 0.001
EDI_Perfectionism	0.132	**0.007**	0.152	< 0.001
EDI_Interpersonal distrust	0.026	0.599	0.129	0.003
EDI_Interoceptive awareness	0.167	** < 0.001**	0.283	< 0.001
EDI_Maturity fear	– 0.030	0.543	0.148	< 0.001

Significant results were highlighted in bold

*EHQ* Eating Habits Questionnaire, *EDI* Eating Disorder Inventory

*The data from the original publication of the E-DOS [22]

### Confirmatory factor analysis

We tested the model fit of the hypothesized one-factor model. Results indicated a poor fit of the one-factor model in general. The three- and the five-factor model using asymptotically distribution-free estimates also revealed a mediocre fit (Table 3).

**Table 3:** Asymptotically distribution-free estimates of the confirmatory factor analysis in PL-DOS: comparison between three models

	PL-DOS one-factor model	PL-DOS three-factor model	PL-DOS five-factor model
*χ*^2^	*χ*^2^(35) = 207.566, *p* < .001	*χ*^2^(32) = 138.226, *p* < .001	*χ*^2^(30) = 156.789, *p* < .001
RMSEA	0.110 (0.095—0.124)	0.090 (0.075—0.106)	0.101
TLI	0.445	0.626	0.524
CFI	0.569	0.734	0.683

As none of the models showed a goof fit of the factorial structure of the PL-DOS, an exploratory factor analysis was run to reveal the factorial structure in the current sample (Table 4).

**Table 4:** Factor loadings of the PL-DOS in an exploratory factor analysis

Item	F1: Negative feelings	F2: Social exclusion	F3: Importance of health nutrition
10. I feel upset after eating unhealthy foods	**0.953**	0.030	− 0.115
6. If I eat something I consider unhealthy, I feel really bad	**0.697**	− 0.151	0.186
9. I find it difficult to go against my personal dietary rules	**0.548**	0.154	0.046
7. I have the feeling of being excluded by my friends and colleagues due to my strict nutrition rules	0.065	**0.841**	− 0.073
4. I try to avoid getting invited over to friends for dinner if I know that they do not pay attention to healthy nutrition	0.023	**0.678**	0.164
8. My thoughts constantly revolve around healthy nutrition and I organize my day around it	0.418	**0.454**	0.044
2. I have certain nutrition rules that I adhere to	0.050	− 0.236	**0.741**
5. I like that I pay more attention to healthy nutrition than other people	0.052	0.119	**0.634**
1. Eating healthy food is more important to me than indulgence/enjoying the food	0.032	0.080	**0.613**
3. I can only enjoy eating foods considered healthy	– 0.022	0.260	**0.523**

Extraction method: maximum likelihood; Oblimin rotation; 68% total variance explained; Kaiser–Meyer–Olkin measure of sampling adequacy was 0.826, Bartlett’s test of sphericity was significant (*p* > 0.001)

Numbers in bold indicate significant p-values

### Prevalence of ON in the Polish university students sample

Of the 412 participants, 6.6% (6.3% of female, 7.4% of male participants) had total scores greater than or equal to 30 indicating ON (according to the proposed 95th percentile), and 11.9% (12.6% of female, 9.6% of male participants) had total scores in the range of 25–29. No risk of developing ON was observed in 81.6% of the study sample (Table 5).

**Table 5:** Comparison of the prevalence of ON among Polish and U.S. students [22]

DOS	Polish university students	U.S. university students
Presence of ON (≥ 30)	6.6%	8.0%
At risk of ON (25–29)	11.9%	12.4%
No risk of ON (< 25)	81.5%	79.5%

## Discussion

The aim of the current study was to examine the psychometric properties of the PL-DOS in the Polish cultural setting. Moreover, the prevalence of ON, assessed by the PL-DOS, among Polish university students was also evaluated and compared with that of U.S. students (assessed by the E-DOS). Our findings indicated that the PL-DOS has good reliability (*α* = 0.840, *ω* = 0.840), nevertheless the CFA with the one-factor model, as well as the three- and five-factor models showed poor model fit. These results are in line with findings from previous studies focusing on examination of the original German version of the DOS [19] and its English version [22] and are opposite to recent research findings evaluating a Spanish (DOS-ES) [23] and a Chinese version of the DOS (C-DOS) [24]. We can suppose that the cultural context (Western versus non-Western cultures) may affect these results (e.g., higher levels of body dissatisfaction are observed in Western society, Chinese people tend to present more self-control [32]; higher prevalence of ON in Chinese males is suprising, as there is a higher prevalence of ED in Chinese female adolescents [33]; the more Westernized the more body concerns and desire to be slim [34]).

The sum score of the PL-DOS positively correlated with all the subscales of the EHQ (problems associated with healthy eating, knowledge of healthy eating and feeling positively about healthy eating), suggesting good convergent validity of the PL-DOS. The total score of the PL-DOS had low correlations with eating disorder psychopathology and related features (drive for thinness, bulimia, interoceptive awareness and perfectionism) measured by the EDI. This indicates the good divergent validity of the PL-DOS and is consistent with the previous results [22–24]. These findings suggest that the construct of ON measured by the PL-DOS varies from the eating disorder psychopathology measured by the EDI [24]. The lack of correlation between PL-DOS and body dissatisfaction, ineffectiveness, interpersonal distrust and maturity fears measured by the EDI could suggest that these features of eating disorder pathology have less in common with the construct that the PL-DOS captures. The finding that PL-DOS is not related to ineffectiveness or interpersonal distrust might be surprising at first glance. In individuals with ON, commitment to follow a rigid diet plan (“pure” dieting) can be caused by an intense need for control to compensate for low self-esteem and feelings of ineffectiveness [35]. Interpersonal disruption is considered as being an essential trait in the assessment of ON with regard to the social isolation that it causes [23]. According to the EDI, ineffectiveness involves feelings of general inadequacy, insecurity and not being in control of one’s own life. It has been described as the fundamental disturbance in AN. Contrarily, interpersonal distrust reflects a sense of alienation and a general reluctance to have close relationships. Given that the meaning of these features strictly refers to eating disorder pathology, the lack of a relationship between the PL-DOS and ineffectiveness and interpersonal disruption measured by the EDI is not surprising.

Our results showed that ON was prevalent among Polish students. Using the PL-DOS, our findings revealed that 6.6% of the sampled Polish university students were classified as having ON and 11.9% were at risk of developing ON. This indicates that the prevalence of ON, based on the PL-DOS, is lower than the prevalence found in the United States (8% of U.S. students displayed ON and 12.4% were at risk of developing ON) [22], China (7.8% displayed ON and 18.2% were at risk of developing ON) [24] and Spain (10.5% displayed ON) [23]. It seems, therefore, that ON and being at-risk of developing ON might be more prevalent in these countries than in Poland or Germany (3.3% of German students displayed ON and 9.0% were at risk of developing ON). The translation of the DOS could be also a potential reason. The Polish version of the DOS was obtained by translating the English version of the DOS and not the original German version of the DOS. Using this indirect way to translate the DOS might have yielded additional inaccuracies in the original meaning of the items. There are cultural differences in the interpretation of many terms, back translation may mask the critical differences between the two versions of the questionnaire, back-translated items may differ from their counterparts in the original DOS in the linguistic form they assume and the meaning they convey, linguistic variety chosen by translators may depend on their cultural level [36]. In addition, cultural differences (e.g., food preparation, parental practices, locally available foods and dietary customs) undoubtedly play a relevant role. In countries, where healthy dietary patterns are in the centre of the public health practice, dietary recommendations based on foods are likely to be more accessible [37]. It is worth pointing out that the 2015 US Dietary Guidelines Advisory Committee (DGAC) identified three dietary patterns that improve diet and share several similarities (e.g., a higher intake of fruits, vegetables, whole grains, nuts, and legumes; a moderate intake of alcohol; a lower intake of red and processed meats, sugars-sweetened foods and drinks). These were the healthy US-style pattern, the healthy Mediterranean-style pattern, and the healthy vegetarian pattern. It could be possible that young people in the United States and Spain are more likely to pay attention to healthy eating and to present a strong preoccupation with it, manifested by the avoidance of all foods subjectively considered to be “unhealthy”. This is an issue for future research to explore.

The present study has several limitations. First, this study has been conducted in one homogeneous sample (Polish university students). Second, the number of male students (22.8%) observed in the present study was disproportionate. Therefore, future research should be conducted among more heterogeneous samples. Third, the use of a longitudinal rather than a cross-sectional design would improve future studies. Fourth, test–retest reliability was not examined. Furthermore, Polish studies should determine the reliability of the PL-DOS measured over time. Finally, the English (not the original German) version of the DOS was used to translate it into Polish. Using this indirect way to translate the DOS might have yielded additional inaccuracies or slight differences in the original meaning of the items. It would be more appropriate to use the original German version of the DOS as a basis for the Polish version.

## Conclusion

In conclusion, the findings suggest that any screening instrument has limitations that should be considered before its use. The DOS is a relatively new tool; however, its construction and the psychometric properties appear to measure the ON construct. The use of the same tool across ON research would enhance the comparability of findings among studies and subsequently increase confidence in the interpretation of findings in the field of ON research.

## What is already known on this subject?

Although the amount of ON measures has increased in the last several years, to date, it remains an open question whether existing ON measures are appropriate for the assessment of ON.

## What this study adds?

PL-DOS has good reliability (*α* = 0.840, *ω* = 0.840) and is an appropriate ON measure for a Polish student population. Using the PL-DOS, our findings revealed that 6.6% of the sampled Polish university students were classified as having ON.

## Figures and Tables

**Table 6:**
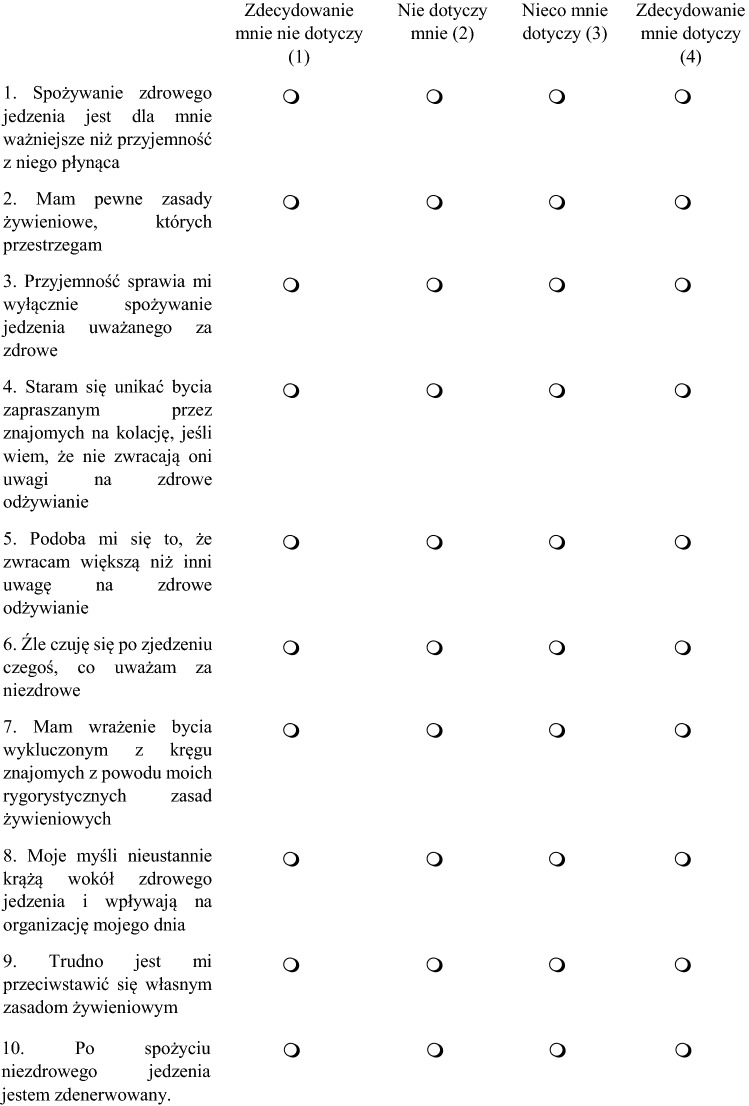
Polish version of the DOS (PL-DOS)

## Appendix 1

See Table 6.
